# Participant recall and understandings of information on biobanking and future genomic research: experiences from a multi-disease community-based health screening and biobank platform in rural South Africa

**DOI:** 10.1186/s12910-022-00782-z

**Published:** 2022-04-18

**Authors:** Manono Luthuli, Nothando Ngwenya, Dumsani Gumede, Resign Gunda, Dickman Gareta, Olivier Koole, Mark J. Siedner, Emily B. Wong, Janet Seeley

**Affiliations:** 1 Africa Health Research Institute, KwaZulu-Natal, South Africa; 2 Division of Infection and Immunity, University College London, London, UK; 3 School of Nursing and Public Health, College of Health Sciences, University of KwaZulu-Natal, KwaZulu-Natal, South Africa; 4 Global Health and Development Department, London School of Hygiene and Tropical Medicine, London, UK; 5 Division of Infectious Diseases, Massachusetts General Hospital, Boston, MA USA; 6 Division of Infectious Diseases, Heersink School of Medicine, University of Alabama at Birmingham, Birmingham, AL USA

**Keywords:** Biobanking, Genomic research, Ethics, Informed consent, Participant experiences, Researcher experiences

## Abstract

**Background:**

Limited research has been conducted on explanations and understandings of biobanking for future genomic research in African contexts with low literacy and limited healthcare access. We report on the findings of a sub-study on participant understanding embedded in a multi-disease community health screening and biobank platform study known as ‘Vukuzazi’ in rural KwaZulu-Natal, South Africa.

**Methods:**

Semi-structured interviews were conducted with research participants who had been invited to take part in the Vukuzazi study, including both participants and non-participants, and research staff that worked on the study. The interviews were transcribed, and themes were identified from the interview transcripts, manually coded, and thematically analysed.

**Results:**

Thirty-nine individuals were interviewed. We found that the research team explained biobanking and future genomic research by describing how hereditary characteristics create similarities among individuals. However, recollection and understanding of this explanation seven months after participation was variable. The large volume of information about the Vukuzazi study objectives and procedures presented a challenge to participant recall. By the time of interviews, some participants recalled rudimentary facts about the genetic aspects of the study, but many expressed little to no interest in genetics and biobanking.

**Conclusion:**

Participant’s understanding of information related to genetics and biobanking provided during the consent process is affected by the volume of information as well as participant’s interest (or lack thereof) in the subject matter being discussed. We recommend that future studies undertaking biobanking and genomic research treat explanations of this kind of research to participants as an on-going process of communication between researchers, participants and the community and that explanatory imagery and video graphic storytelling should be incorporated into theses explanations as these have previously been found to facilitate understanding among those with low literacy levels. Studies should also avoid having broader research objectives as this can divert participant’s interest and therefore understanding of why their samples are being collected.

**Supplementary Information:**

The online version contains supplementary material available at 10.1186/s12910-022-00782-z.

## Background

Biobanking of human samples for future use raises several challenges for informed consent, data and sample sharing, privacy, and sample ownership [1]. These challenges can be particularly severe when conducting human sample collection in lower- and middle-income countries (LMIC) [2]. Explaining what biobanking is and the processes involved in the collection and storage of samples is essential for research participants to make an informed decision about their participation [2, 3]. Previous studies in Africa and elsewhere have found it challenging to explain certain terminologies about the research methods, unfamiliar scientific concepts, the implications of the complex storage and sharing infrastructure required and the possible consequences of future research with the samples and data [2, 4–6]. These challenges are inherent in studies that involve biobanking where specimens can be used for future genomic research [7].

Broad, narrow and dynamic consent strategies have been proposed to address challenges in obtaining informed consent for biobanking and genomics studies [5, 8]. Broad consent is be defined as consent for a non-specific range of future research purposes subject to a few restrictions in terms of content and processes [9, 10]. Broad consent is used when the researcher does not anticipate having time or resources to reconsent and when there is not sufficient risk to the participant from future research that would necessitate reconsent. Narrow or focused consent is for a clearly specified and usually singular research purpose and researchers have to seek additional consent should they wish to use samples for a purpose that previously was not consented to by the participant [9, 11]. Dynamic consent involves the use of physical or digital platforms for on-going communication with participants so they can re-consent to the use of their samples for various procedures. This has been proposed as an alternative but may face challenges in LMIC settings where access to transport and technology may be limited [12].

Recent literature where biospecimens are banked for future genomic research in Africa has seen a growing acceptance of and preference for broad consent [5]. This is due to the challenges associated with narrow consent, including that modern multi-omic approaches rarely follow a linear path and the difficulty and costly nature of reconsenting participants for each new scientific question [11]. The future use of biospecimens is not always clear at the point of consent and seeking consent in the future may be challenging due to the cost and time implications of reconsenting participants [10, 11]. On the other hand, this lack of clarity around the future use of samples in studies conducting biobanking for future genomic research has also led to questions around broad consent as best practice because it can leave participants with an unclear understanding of what their samples will be used for [12].

Proponents of broad consent argue that it is the best consenting strategy in research which involves biobanking because it can cope with the large scale of research and repeated data collection [10]. For broad consent to be applied appropriately, a certain level of trust ought to exist between the researcher and participants and appropriate governance and community engagement structures must be in place throughout the study [13].

Low levels of general, scientific and health literacy in LMIC has made the informed consent process challenging as research participants struggle to recall and understand elements of the process [14–16]. Studies in many different settings report that a majority of participants do not have a background knowledge about or understanding of genetics, which impacts on their comprehension of the information shared with them during informed consent [15, 17, 18]. The complex terminologies involved in describing studies conducting biobanking and future genomic research make recall and understandings of informed consent in this type of research even more challenging [4, 6, 19]. The lack of genetics knowledge among the general population in many places compounds this challenge and yet understanding concepts such as genetics, data storage and sharing, and genomics is necessary for participants to give valid consent [20, 21].

Cognisant of the challenges related to participants’ understanding biobanking and attempting to account for future genomic research, researchers have tended to make Informed Consent Forms (ICF) more detailed and longer, but this has inadvertently raised more questions about how much information individuals can understand and retain to provide informed consent [22]. Language barriers play an influential role as some words and terminologies do not have a local language equivalent so explaining the terms may be difficult for researchers [23]. Previous studies in in low-and middle-income countries, have thus recommended the use of innovative means of seeking consent such as using audio or video tape to capture verbal consent, and preceding individual consent with acquiring community level approval to be inclusive of those that can neither read nor write [17, 24].

The Africa Health Research Institute (AHRI) developed the Africa Centre Demographic Information System (ACDIS) in 2000 to monitor and describe the health, social and demographic impacts of the rapidly growing Human Immunodeficiency Virus (HIV) epidemic and it’s intervention strategies in rural South Africa [25]. The ACDIS was renamed the Population Intervention Programme (PIP) in 2017 as it now covered a variety of infectious diseases and non-communicable diseases (NCD) with the surveillance area covering 845 km^2^ and 140,000 individuals in 20,000 homesteads in UMkhanyakude District, KwaZulu-Natal [25].

In response to increasing prevalence of Tuberculosis (TB) and NCD and increasing focus on biomedical science, AHRI developed a multi-disease screening study which included a specimen biobank in 2018. This study was known as Vukuzazi which means “wake up and know yourself” in the local IsiZulu language. The aim of Vukuzazi was two-fold: (1) to conduct health screening to determine the population-based epidemiology of HIV, TB and NCD’s epidemics in uMkhanyakude District; (2) to collect and store biological specimens with a view to performing cutting edge science and genomic research to yield insights into the biological interactions and health implications of these intersecting epidemics in uMkhanyakude District [26, 27]. Using broad consent as the sole consenting strategy, Vukuzazi deployed mobile health screening camps throughout the surveillance area which sought to screen for the most significant infectious diseases and NCD’s, referring newly diagnosed cases for treatment, collecting, and storing biological samples, and supporting novel research on the relationship between host, pathogens and NCD’s [26].

Explaining and understanding biobanking and future genomic research was an intrinsic part of engagement between Vukuzazi research staff and research participants. The AHRI team had limited experience in providing such information. Indeed, there has been limited research conducted on how biobanking and future genomic research is explained and understood by people in resource limited settings in Africa, particularly those with low literacy [15].

Through a qualitative methods sub-study of Vukuzazi, we sought to explore participant understandings and perceptions of the Vukuzazi research objectives and procedures as well as the participants’ reasons for participation and non-participation. This qualitative sub-study was important to understand participant impressions of a study like Vukuzazi and gauge satisfaction or areas of concern. In a recently published article from the same study by Ngwenya et al. [6], results showed that participants had a difficult time understanding the biobanking and future genomic research objective of the study. Therefore, in this paper, we focus more closely on the findings of this qualitative methods sub-study which highlighted participant experience of recalling and understanding biobanking research in a rural South African context.

### Study setting

This study was carried out in uMkhanyakude District, in the northern part of the KwaZulu-Natal Province in South Africa. This region is the second poorest District in South Africa with the majority of the population living below the poverty line [28, 29]. There are low levels of employment with 31 percent of the overall population being unemployed, and only a fraction of these unemployed people have completed tertiary level education [28]. Furthermore, low school education levels have resulted in low adult literacy levels with more than 22 percent and 27 percent of the adult male and female population respectively, not receiving any form of school education [28].

The region is largely under-resourced in terms of healthcare facilities as there are only five district hospitals and fifty-seven clinics servicing a population of over six hundred and twenty-five thousand, spanning over twelve thousand eight hundred and eighteen square kilometres [30].

## Methods

### Study aim, population, sampling, and participants

The data for this paper were collected through the Vukuzazi qualitative methods sub-study. The overall aim of this sub-study was to gain insights into participant understandings and perceptions of the Vukuzazi study and its procedures—biobanking and future genomic research were two of the study procedures. To get a well-rounded sense of the experience of explaining biobanking and future genomic research, we targeted the entire population of people who were eligible to participate in Vukuzazi and the staff that worked on the study. This population was part of the AHRI population surveillance platform which includes members of households within the community that usually participate in AHRI research studies [25].

To produce a random sample from the population of all the people who were eligible to participate, a statistician used a computer algorithm stratified by participation, non-participation, sex, age, varying conditions of health and disease. Table 1 below represents this sampling criteria. This sample would make up all the participants of the qualitative sub-study which sought to understand participant impressions of the Vukuzazi and investigate any areas of concern. The sample of staff members represented the different positions that people worked in in the study—this was a convenience sample. See Table 1 below for the sample selection criteria:

**Table 1:** Sample selection criteria

Gender	Sample				
Males	3 non-participants [one under 24 years, one 24–50 years, one over 50 years]	3 with either new active TB or new HIV [one under 24 years, one 24–50 years, one over 50 years]	5 with multimorbidity (i.e. with two of either HIV, active or prior TB, Diabetes, Hypertension) [one under 24 years, one 24–35 years, one 36–50, two over 50 years]	4 with no disease [one under 18 years, one 18–24, one 25–45 years, one over 45 years]	Must have received first round of results
Females	3 non-participants [one under 24 years, one 24–50 years, one over 50 years]	3 with either new active TB or new HIV [one under 24 years, one 24–50 years, one over 50 years]	5 with multimorbidity (i.e. with two of either HIV, active or prior TB, Diabetes, Hypertension) [one under 24 years, one 24–35 years, one 36–50, two over 50 years]	4 with no disease [one under 18 years, one 18–24, one 25–45 years, one over 45 years]	Must have received first round of results

### Study participants

The participants consented to participate in Vukuzazi in May 2018 and then seven months later, some of them were recruited to participate in the Vukuzazi qualitative sub-study from which the data for this paper was obtained.

The sub-study sample included participants and non-participants as well as staff members that worked in Vukuzazi. Three female and five male staff members (n = 8) had an age range of 24–41 years; five male and two female (n = 7) non-participants with an age range of 24–52 years; and nine male and 16 female participants (n = 24) with an age range of 22–75 years made up the total number participants (n = 39) that took part.

### Data collection

Data were collected through semi-structured in-depth interviews (IDI’s) focusing on participant views of the Vukuzazi study, including their perceptions and understandings of biobanking and the future genomic research on biospecimens. The other topics included reasons for participation and non-participation, knowledge, understandings, and perceptions of other parts of the study like informed consent, health screening, the recruitment process, and the return of results—these topics were necessary for the wider qualitative sub-study but they will not be included in the results section of this manuscript as they fall outside the focus of the manuscript title.

Participant’s contact information for the qualitative sub-study were extracted from the PIP database for the purposes of recruitment via telephone calls. The interviews were conducted by a trained Senior Social Science Research Assistant in the participants’ preferred time, location and language (isiZulu or English). The study was explained in detail to every participant when they were recruited and before the commencement of the interview. Each interview only began after the participant read through the information sheet, had an opportunity to ask the interviewer questions and then signed the ICF. All interviews were audio recorded using a voice recorder. The interviews ranged from approximately fifteen to sixty minutes in length.

The seven months’ lapse between data collection in the Vukuzazi study and our sub-study which involved assessing comprehension was mainly due to the convenience of the sampling and access to participants willing to take part in an interview. The participants were contacted after they completed participation in the Vukuzazi study and had received their clinical test results which sometimes took a couple of months and then arranging a convenient interview appointment also took sometime.

### Data management and analysis

Audio files of each interview were electronically stored in an online server-based drive within a folder to which access was restricted to the staff members working in the qualitative sub-study. All the audio files were transcribed into English by the same Senior Social Science Research Assistant that collected the data. The transcripts were checked for accuracy by a Senior Social Scientist who listened to the audio files whilst reading the transcripts. All the transcripts were de-identified, with all identifiable data removed to ensure anonymity and confidentiality.

Reflective field notes were compiled into a formal interview summary document as an initial phase of data analysis whereby key emerging points were grouped into themes and sub-themes. These summaries also detailed non-verbal events occurring during the interview which could provide additional insights into the content that emerged from the interview.

The field notes and the transcripts were also saved onto the online server-based drive in a folder that had access restricted to the staff members working in the qualitative sub-study. An iterative process of data collection and analysis was utilised where transcripts were reviewed after each interview to inform the data collection. Transcripts were reviewed and discussed by a team of two Social Scientists and the Senior Social Science Research Assistant to inductively form the coding framework. The process involved categorising data into codes which were discussed and later grouped into themes aligned with the research objectives. The coding framework was critically reviewed for accuracy and inclusiveness throughout the duration of the data analysis process—this facilitated the process of organising the data and extracting the emerging themes. All coding of transcripts was done manually, following the principles of reflexive thematic analysis. Furthermore, data analysis was guided by a phenomenological research design as we sought to understand participant’s lived experiences of recalling and understanding biobanking and genomic research.

## Results

We grouped our findings on the experiences of how biobanking and future genomic research was explained and understood in this study under three sub-headings: (1) the way the research team explained biobanking research; (2) what participants said they understood from this explanation; and (3) how participants think biobanking and future genomic research should be explained to people in this context.

In total sixty-seven people with ages ranging from 22 to 75 years-old were invited to participate in the qualitative sub-study; specifically eight staff members, twenty people who had declined to partake in Vukuzazi and 39 who had participated in Vukuzazi. A total of thirty-nine people accepted our invite to participate in the study so our results were produced from thirty-nine interviews conducted over an eight-month period with twenty-four individuals who participated in Vukuzazi, seven of those who did not participate in Vukuzazi and eight members of the research team that worked in the study.

### The way the research team explained biobanking and future genomic research

The ICF was the primary source of information for participants and the research team’s explanations were mainly a means of consolidating what is in the ICF by talking through its contents to provide emphasis and to provide clarity for those who did not understand the contents or may not be able to read. All explanations were provided in the local language of IsiZulu. A clinical member of the research team insisted that participants do receive an explanation about biobanking and genetics, but some end up forgetting because they do not give themselves the necessary amount of time to listen:

*They get an explanation. There are some people that need re-explanation when the follow-up team comes around because they were in a rush when they got the first explanation and didn’t quite get what was said* (Participant Twenty-three).

However, some participants claimed that they were only told that their blood samples would be used for health screening purposes, and they did not know of anything more than that. A participant claimed that the research team *“didn’t explain anything to me regarding the blood samples.”* (Participant Six).

Furthermore, in response to a question about whether they explain the genomic research component to participants and why some of them cannot recall or understand it, a clinical member of the research team stated that some participants were not interested in any explanations because they just want the grocery voucher which was provided for taking part in Vukuzazi:

*They understand what Vukuzazi is all about although there are young people whose purpose isn’t to know about the state of their health, you explain everything to them but you can see that they aren’t really interested because they are just there for the voucher.* (Research Team Member Five)

It is important to note that the informed consent process unfolded in the following manner, as described by a old male clinical member of the research team:

*Upon arrival at the community-based health screening platform, participants are seated in groups of ten to twenty and they are given an ICF to read through which provides full details of all study procedures including the biobanking and genomic research component. Thereafter, a member of the research team explains the contents of the ICF and gives participants an opportunity to ask questions regarding the ICF—roughly fifteen to thirty minutes is allocated for participants to read, ask questions and think about whether they want to participate. Prior to the participant’s arrival and as part of the recruitment and consenting process, participants receive a detailed initial explanation about the study and its procedures when the research team visits their homes to recruit them.* (Research Team Member Five)

Another clinical member of the research team confirmed that they do not usually go into great detail when explaining the biobanking and genomic research component of the study, but they tried to explain genetics by referring to similar characteristics shared among family relatives as well as different responses to treatments among people with the same disease:

*What we tell them is that there is a blood sample that we are going to use to test their genetics and they know that they won’t get the results of these tests because the research is still under way. We give them an explanation if they ask for one. We explain that sometimes you may have an illness and there is a relative or a family member that has it too and you are both on treatment for that illness, but your bodies don’t respond to it the same way. The difference in response is due to our differing bodies and genetic make-ups.* (Research Team Member Six)

She went on to divulge that when people did not understand they tried to further explain by talking about hereditary features and characteristics among family members:

*Some people don’t really understand what genes are even when we explain it to them. We try to explain it by saying that there are some diseases that just run in the family—like diabetes—one might be surprised to learn they have diabetes, not knowing that someone in their family has previously had it too. I also explain it to them by making an example of a couple getting into fights because the wife gives birth to a light skinned baby, yet the husband is dark skinned and thus believes the baby is not his. It may very well be that someone in their family from previous generations had those light skinned genes and the new-born baby took after them.”*

The study’s objectives, including the biobanking aspect, were explained during a participant’s recruitment, upon their arrival at the research site and at each individual station within the site. All these explanations were given by different team members with different team functions at each point and according to a clinical member of the research team, explanations varied in the detail provided:

*People are different, some explain clearly what was told to them at home but sometimes we find that the information they got at home was inadequate. As nurses it is also our job to explain the purpose of the study again because we are the ones who understand it better than the recruiters. In terms of percentages, most of them arrive knowing what it is all about. They give their consent knowing exactly what they are consenting to because there are also various talks given at the camp site before they get to us the enrolled nurses.* (Research Team Member Five))

The different types of explanation required at the screening points (referred to as stations), where different types of tests were done or measurements taken, caused some confusion as was observed by Participant Nine:

*But there were some stations at which I didn’t get an explanation—like at the measurements station—they just told me they would be measuring this and that and then you will go to that station. When I got to that station, they just said we will be taking blood samples into all of those tubes.*

### What participants said they understood and recalled from this explanation

Overall, participants’ recollection of explanations related to genetics and biobanking was limited, possibly because of the time which had elapsed (7 months) between them being consented to join Vukuzazi and them being asked to recall what they were told in our sub-study. Participant Twenty-One, for example, when asked about how genetics and biobanking was explained to her, attributed her lack of recollection to the length of time that had passed:

*I can vaguely remember them mentioning something about DNA but I can’t quite recall the context in which it was mentioned. It was a long time ago, you will have to forgive me. I think they just said they would be observing how our genes behave. That is all I can remember. I’m sorry.*

Participant Ten narrated her account of what was explained to her regarding genetics and biobanking but ultimately admitted that she remembered some but not all of the details that was explained to her:

*I don’t remember accurately. I remember one of the guys I lived with asked him about some results and the AHRI nurse said they would come back in future because they are still waiting for the right machines to process them. I forgot what illness she said that is. Yes, she said the machines have not arrived but I can’t remember what illness that was. […] but I can’t quite remember anything about DNA related stuff you know.*

In response to a question about her understanding of what the study she participated in was about, Participant Fourteen said that could not remember the exact details of what was discussed with her because of the large volume of information that was explained to her during the consenting process. Referring to the staff member who gave her the explanation, this woman noted that: *“She spoke quite a lot. I don’t remember what she said exactly. But she definitely did explain. She didn’t hide anything.”*

When asked about why participants had such poor recollection and understanding of the genetics and biobanking component of the study, Participant Twenty-Three revealed that a lot of participants are probably not interested in this part of the study, hence they do not pay much attention when it is being explained:

*Some probably aren’t interested in hearing about this. How can I put this, some people just aren’t interested in knowing about their state of health anyway. They are just ok with not knowing themselves. They just go there because they heard they will get something in return. Some people are just there for the vouchers. Not because they genuinely want to wake-up and know themselves like the name of the study says*.

On the other hand, when asked the same question, participant Twenty-Two alluded to the fact that the complexity of certain terminologies and concepts of genetics and biobanking would be difficult to understand for most people. However, she went on to note it is possible to understand if one really makes an effort:

*…it is complicated when you consider the many factors that are involved. The family members, the illnesses, the types of medication and how all these factors relate in this entire process. People often just choose to believe that medication fails to work because it just doesn’t work - that is just the easiest thing to believe. I think if one listens to the explanation properly and they want to understand, they can understand*.

Participant Twenty-Four noted that some people may not understand or recall the genomic research component of this study because “*some people don’t believe in these things. These DNA things, these scientific things. There isn’t much I believe in. I’m sceptical about a lot of things.*”

Considering that there were certain participants with limited literacy levels, there was significant variation in people’s ability to understand genetics and biobanking. This was evident in Participant Thirteen’s response to a question about what she understood and recalled from the information sheet and consent form she said *“I understood that nothing was compulsory so if you didn't want to do something you could just say so. I signed with a cross because I don't know how to read.”*

How participants think the consent process should have been conducted to facilitate better recall and understanding of biobanking and genomic research.

The participants felt strongly about the fact that all parts of the study, including biobanking and genomic research, should be explained slowly and thoroughly while giving people the opportunity to ask questions—as was discussed by Participant Twenty-Four:

*I think as a Zulu person yourself you know that our people tend to be stubborn. They want things to be explained thoroughly and fully. They want to be made to understand that doing this will eventually benefit them and future generations. They need to be shown how this will all happen. People need things explained slowly and they must have the freedom to tell you if they don’t understand. Remember at some point during your explanation I told you that you didn’t make sense? And then you put it differently.*

He went on to allude to how the delivery of explanations regarding information related to the study is the most important part of the work any researcher does, particularly when doing research that involves biobanking and future genomic research because it is more complex and difficult to understand:

*That’s what is most important, people doing the sort of work you do (explaining biobanking and genomic research) need to be very patient and clear in their delivery of explanations. They should take their time and the problem is sometimes they are in a rush to finish so they can go do their own things. Patience would really help.*

When asked about how best to explain genomic research to participants such as those in this context (with low income and low literacy), Participant Twenty-Two suggested that an emphasis on hereditary characteristic should be made:

*What can I say… you can explain it by saying that the research looks at hereditary characteristics in your family and how they relate to illness that one has or might be susceptible to. It’s not necessarily that you got infected. Yes, the ‘hereditary’ concept needs to be emphasised.*

When an explanation describing genomic research in the context of hereditary characteristics and their relation to illness susceptibility was given to Participant Ten, they acknowledged that explanation was good: *“I think the way you explained it was good.”*

In summary, the findings of this paper demonstrate a mutual interconnectedness between the experiences of explaining and understanding biobanking and future genomic research. Our exploration of participant’s understanding of biobanking and future genomic research in our context conveyed two key messages: firstly, participant’s understandings of the biobanking and genetics explanations were affected by their interests in the study’s broader health screening agenda. Secondly, the researcher’s ability to slowly explain and break down scientific concepts in lay terms, without being affected by the participants interest or lack of interest and whilst giving the participants time to ask questions, directly affects how biobanking and future genomic research is understood.

## Discussion

Our findings build on the paper written by Ngwenya et al. [6] which found that participants had a difficult time recalling and understanding biobanking and future genomic research. It was important to build on the manuscript published by Ngwenya et al. because it evaluated participant understandings of the whole informed consent process but it did not look very deeply into how and why recalling and understanding biobanking and genomic research was so difficult for participants. We revealed diverse and rich experiences from researchers who were explaining biobanking and future genomic research and also the participants on the receiving end of these explanations. Similar research, although limited, has been done in other contexts so this manuscript is important to compare and contrast any similarities between experiences in this context and others.

In Vukuzazi, researchers had to explain the biobanking and future genomic research component of the study in addition to explaining all the disease-screening elements, which meant that the consenting process was lengthy, with an information and consent form which was almost 14 pages long. Some participants in our sub-study highlighted the volume of information they had to consume as a key factor in their lack of recall and understanding of some parts of the study, including the genomic component. Findings from previous research studies have revealed that providing participants with a lot of information at once can result in poor recall and understanding of some aspects as participants inadvertently filter out some information, they deem not to be important or because they cannot determine what is important to retain and what they can disregard [22, 31–33].

Furthermore, genetics and biobanking has been found to be of less interest and subsequently poorly recalled and understood by participants partaking in studies with a focus that is broader than just biobanking and genomics [3]. This is consistent with findings in our study. We found that participants showed a much clearer understanding and recall of the disease-screening portion of the study along with other benefits, such as the grocery voucher—factors more aligned with their interests and needs [3, 34]. The participants trusted that they had participated in AHRI research previously and it had always been harmless and very beneficial [35–37].

Vukuzazi staff used simplified and pre-rehearsed stories about hereditary characteristics related to physical appearance and illness, which has been found to be effective in other studies with complex terminologies [38]. These explanations were provided at different points in the study (during recruitment, during informed consent procedure and during blood sample collection) but our findings show, that this information was not recalled and understood well by all participants. This is similar to past studies in low-income settings as researchers have found explaining biobanking and future genomic research to participants to be a repetitive and lengthy task due to literacy levels, lack of background knowledge of genomics and the limitations of local languages for explaining such concepts [1, 7, 17, 33, 39]. Past studies have recommended that researchers undergo specialised training on explaining research objectives and obtaining consent prior to commencing data collection in any study conducting biobanking and future genomic research with human participants [15, 31, 33].

Overall, participants of our sub-study felt that even if participants exhibit a lack of interest and understanding of genomics in any study, it is important for researchers to continue to explain in detail if they wish to acquire participant’s consent [33]. The use of simplified and pre-rehearsed stories about hereditary characteristics and illness in family was endorsed by participants. Participants in our study suggested that to make these explanations most effective, researchers ought to be prepared to engage in on-going dialogue whilst applying patience, slow explanations, a willingness to let participants reflect and ask questions, and to let them consult with a friend or relative when necessary—some of this is consistent with what other studies have found [40–42].

As many of our findings are consistent with what has been found in similar resource limited settings such as African LMIC’s, this study consolidates the existing literature by emphasising just how common participant struggles with understanding and recalling genomics are, hence current and future studies will thus have further grounds to plan for this. Previous similar studies, along with ours, also provide sufficient insight for scholars in terms of how exactly to plan for navigating the challenges related to participants’ understanding and recall of biobanking and genetics information.

A key limitation of the study was the heavy reliance on what the clinical staff said they did during the consent process with no direct observations of the process to provide evidence to substantiate what was reported.

## Conclusion

Explaining the Vukuzazi research objectives was a crucial part of engaging with the participants and the wider research community in the requisite ethical manner [6]. We recommend that to enable participants to better understand biospecimen collection and future genomic research, future studies involving biobanking and genomic research in settings with low literacy and limited healthcare access, should ensure that the explanations of biobanking and future genomic research are treated as an ongoing process of communication between researcher and participant rather than as a once-off event.

We also recommend giving explanations that utilise audio and visual story telling through pictures and videos in native language as this has been found to make complex concepts and information easier to understand for those with low literacy levels [31, 33]. Such explanations should draw from people’s own knowledge of hereditary characteristics, as well as stressing the benefits of planning for future research. It is important to engage participant’s interests and thus enhance their understanding.

## Supplementary Information

**Additional file 1.** Participant Information Sheets and Informed Consent Forms. Description of data: Vukuzazi Participant Information Sheet.

## Data Availability

The data underpinning this article cannot be shared publicly due to privacy and the potential identification of participants in the study. Data is available from the AHRI Research Data Management committee (contact via RDMServiceDesk@ahri.org) for researchers who meet the criteria for access to confidential data. The datasets used and/or analysed during the current study is available from the corresponding author on reasonable request.

## Abbreviations

ACDIS: Africa Centre Demographic Programme Information System
AHRI: Africa Health Research Institute
HIV: Human immunodeficiency virus
ICF: Informed consent forms
ICT: Information Communications of Technology
IDI: In-depth interviews
LMIC: Lower- and middle-income countries
NCD: Non-communicable diseases
TB: Tuberculosis
