# Supplementary material for: Participant recall and understandings of information on biobanking and future genomic research: experiences from a multi-disease community-based health screening and biobank platform in rural South Africa
Source: BMC Med Ethics. 2022 Apr 18;23:43. doi: 10.1186/s12910-022-00782-z (PMC9014601; doi:10.1186/s12910-022-00782-z)
Supplement: Supplementary file 1 — Additional file 1. Participant Information Sheets and Informed Consent Forms. Description of data: Vukuzazi Participant Information Sheet. [file 12910_2022_782_MOESM1_ESM.docx]

**Appendix A: Participant Information Sheets and Informed Consent Forms**

**Information Sheet and consent form - ALL 15y AND OVER**

(If participant is aged under 18, parent or guardian must sign to indicate their consent to the child’s participation in the study, except when child is emancipated)

To be provided to, and discussed with the following people:

- 18 years old or above
- 15-17-year olds when parent or guardian available (parental consent and then child assent)
- 15-17-year olds living alone without parent or guardians


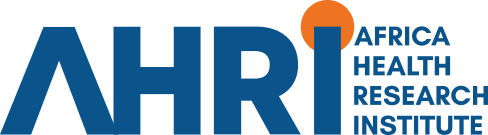


# POP-C/ Vuk'uzazi

# *Participant Information Sheet*

*You are being invited to take part in a research study. Before you decide whether or not to take part, it is important for you to understand why the research is being done and what it will involve.*

*Please read the following information carefully and discuss it with others if you wish. Ask us if there is anything that is not clear or if you would like more information. Thank you for reading this.*

1. **What is this research study about?**

We want to collect information on important health problems affecting your community. This will help us to better understand health and disease in South Africa. We would like to know how common diabetes high blood pressure and cancer are, as well as infectious diseases like HIV and tuberculosis.

We also want to learn how to diagnose these health problems better and to understand why different people's bodies have different responses to infections. Using the information, we collect in this study, we can help to plan future health care to prevent and treat diseases affecting your community.

1. **Why have I been invited?**

You have been invited to take part in the study because you live in the study area where AHRI is active. We will be inviting about 50,000 adults in total from across uMkhanyakude District to take part in the study.

1. **What will happen to me if I decide to take part?**

Your participation in the study will involve:

- An initial visit to **a mobile clinic** and **mobile X-ray team,** very close to your home.
- If any of your results need to be repeated or disucssed, you may receive a **nurse follow-up visit** in your home, 2 or 3 weeks after your visit to the mobile clinic.
- Linkage to existing **medical records** .
- Referral for care at the clinic if you require it.

**Initial visit to our mobile clinics**

In your initial visit to the **mobile clinic team** we will:

- Ask you a series of **questions** about your health and lifestyle (for example, your diet, physical activity, tobacco use, alcohol consumption and medical history). This will last around 30 minutes.
- Record your **height, weight, waist hip and arm circumference, and blood pressure**. These results will be explained to you, and you will be told if you need to seek further advice.

At this visit we will also collect the following samples:

- **Blood samples** (up to 45 ml, equivalent to three tablespoons) - these samples will be collected from your arm by a trained member of the study team, using only sterilised needles. The samples will be examined for diabetes and HIV. Some of the blood will be used by the research team to try to discover better diagnostic tests and to answer questions about the differences between people's bodies.
- A **urine sample which you will collect yourself.**
- A **rectal swab** which you can collect yourself or a nurse can help you with.

The mobile clinic team will be followed by a **mobile X-ray team**. They operate in a separate mobile clinic with a radiology machine and the clinic is staffed by a nurse and radiographer.

Because tuberculosis is a common problem in the community, we will ask you for signs and symptoms of tuberculosis, and we will invite you for a radiography of the chest (except for when you are pregnant). To make sure you are not pregnant, we will take a urine sample from women aged from 15 years to 49 years and do a pregnancy test, before the X Ray.

If we find symptoms or an abnormal "spot" on your chest x-ray, we will ask you for a sputum that we will examine for the presence of TB germs. If you cannot produce sputum we help you breath in a clean salty mist to help make your body produce a cough reaction and sputum.

**Results of your tests**

At the mobile clinic and x-ray visits we will explain all the tests to you and what the test results may mean. On the day, you may be given blood pressure results and also the pregnancy test result. Other tests are sent away (including your chest X ray pictures, for a careful check by a doctor). We will confirm with you which of these test results you would like to be told about. These tests may take 2 to 4 weeks to process. If all your results come back showing that you are healthy, we will provide you with these results by SMS (if you have a cell phone) or telephone.

**Home follow-up visit by a nurse**

If we find any results that are not normal, 2 to 4 weeks later we will contact you to arrange a visit to your home. These abnormal findings include a new positive HIV test (not previously known), raised blood sugar, an abnormal chest X ray, or the presence of TB germs in the sputum. While at the mobile clinic, we might also have arranged to visit you at home if your blood pressure was high, to take further measurements.

During this visit, we will inform you of any test results that are important for your health. These include

- An HIV positive test
- A positive TB test
- If we know you are in HIV care, but your virus level is not controlled
- An abnormal mark on your chest X Ray

Depending on your results so far we may recommend:

- A repeat blood pressure measurement
- A fingerprick test for diabetes

We will arrange referral for any further care that you may need (for example, if you have been found to have HIV or diabetes).

**Linkage to medical records**

We will also ask you if we can look at your personal primary care and hospital records to help us understand your results better and to inform your usual clinic about any new tests sent during this study. This will be done only with your consent. Any information collected will be strictly confidential.

1. **Why are these samples collected?**

Diabetes, high blood pressure, and other related diseases are becoming increasingly common in South Africa. Most people are unaware that they have these conditions. Evidence of these diseases may be found in the blood. We can also look for infectious diseases in the blood and urine. We want to know how many people in this area have these and other related conditions. The good and bad germs that live in our body can also play a role in how healthy or sick we are. These germs can be identified in our stool or on a rectal swab. We want to study how these germs may influence health and disease in our community.

We will also use your blood to study something called “genes”. These “genes” are present in all of us and are responsible for why people in families look like each other. This kind of information is passed from parents to their children and helps to decide for instance how tall you will be and what your body shape will be. Some of these genes may prevent us from getting sick and some other genes may be one of the reasons we get sick when others do not. Studying genes along with health information will help the researchers better understand what causes certain diseases in our community.

1. **What will happen to the study results and the samples I give?**

**Blood, sputum and urine samples**

We will send all your blood, sputum and urine samples to an accredited laboratory where they will be analysed. Your samples will be labelled with a unique study ID number, so that your name will not be linked to your samples and you cannot be identified.

If the initial test for diabetes is negative, you will be informed by phone or SMS. If the initial test for diabetes is positive, you will be visited again for another sample.

If the sputum is negative for tuberculosis, you will be informed by phone or SMS. If the sputum is positive for tuberculosis, you will be referred to a DOH clinic.

**HIV from blood**

We will test your blood for the presence of HIV. At the time of the test you will be counselled so that you understand what to do and how you may feel if your test is positive or negative.

Everyone has a right to know their HIV status and knowing your status will positively impact your health whether you are negative or positive. If, however, you do not wish to learn your HIV status at this time, you have the choice not to be informed of your result. If you choose to receive the HIV test result and the test is negative, you will be informed by phone or SMS. If you choose to receive the HIV test result and the test is positive, a nurse will visit you to inform you about the positive result, discuss your feelings, and refer you to HIV care.

If you are found positive we will also do a CD4 cell count (to check your immunity) and a viral load (to see how much of the HIV virus is in your blood). These results will be discussed with you during the home follow-up visit by a nurse.

If you chose not to be informed of your HIV test results, you will get no information on the HIV test result, whether it is positive or negative.

**DNA from blood**

To look at genetic material, your samples may be sent to Africa Health Research Institute (AHRI) in South Africa. Your genetic material may also be sent to other research institutions, service providers and commercial organisations in these and other countries, to carry out some of the study procedures. Because the long-term effects of the genetic differences are not known, we will not provide the results of these tests to you. If scientific developments occur and something we find in your genes could help your medical care in the future, we will contact you at that point and explain what we have found and how it might be addressed.

**Rectal swabs**

The kind of germs that live in our bowels might have an influence on our health or the diseases we might develop in the future. The rectal swab will be used to look at which bacteria live in your bowels. This is a new area of science and because the effects of these differences are not understood well at this point, the results of these tests will not be provided to you.

1. **How will you follow me up in the future?**

After you have taken part in this study, we may contact you to get your opinion on what it was like to participate in the study. We would also like to keep in touch with you over the next five years. This is because we are interested in finding out whether people develop certain diseases, and whether information we have collected at this visit can help us predict who might be at risk of certain diseases. The diseases we are interested in are: diabetes, heart disease (heart attack and heart failure), stroke, tuberculosis and HIV.

That is why we ask you if we can contact you to participate in future studies.

1. **How will the information and samples that I give in the study be kept private?**

To protect your privacy, we will use a unique study ID number to identify you and all personal information about you, including your samples. Under no circumstances will your name, address or telephone number be passed onto anyone without your consent. However if you are wishing to be given results, we will keep a record of your phone number in case one of our nurses needs to contact you.

All personal information, clinical information and samples we collect will be kept securely locked. Only authorised members of the research team will have access to your personal information and samples. Your name or any other identifiable information will not appear when we present this study at scientific conferences and meetings, or publish our results in scientific journals.

All the results from laboratory testing will be kept confidential. The information linking your name to the unique study ID number will be locked securely with strict access control and will not be made available to the researchers working on your samples. Your name or any other facts that might point to you will not appear when we present this study or publish its results.

If you are diagnosed with TB as part of the study, we will inform the Department of Health. According to the South African guidelines, all new TB diagnoses must be reported. This ensures that people with TB get the treatment they need and that their families get checked for TB.

The District Department of Health has requested access to Vukuzazi test results so that they can best plan for the healthcare needs of the community. If you agree, we will transfer the HIV, blood pressure, and diabetes results and your identifying information to the District Department of Health in the most secure manner possible. You may participate in this study whether or not you agree to share this information.

1. **What are the possible risks of taking part in this study?**

We do not expect that you will get sick or unwell from this research.

When you give blood, the risks to you are small. You may get some slight bruising or bleeding where the blood is taken from your arm. If you experience any discomfort, it will most likely only last a short time (1-3 days).

Some people, who have symptoms that could be due to tuberculosis or who have an abnormal spot on their chest x-ray will be asked to cough up a sputum sample. When the nurse administers saline solution to help make your body produce a cough reaction, you may become uncomfortable or experience mild wheezing. This will be minimized by having trained and qualified staff conducting the procedure and inhaled medications will be available should you experience significant wheezing. If you have asthma or are having trouble breathing already, we will perform your sputum tests at the clinic where it will be safe for you.

As everyone’s genetic information is unique to them, there is a small, theoretical risk that you may be identified from the data produced by this study. We believe that this risk is very small, but you need to be aware of this possibility that you could be identified by your genes someday.

1. **What are the possible benefits of taking part in this study?**

People can have diabetes, high blood pressure, HIV and TB for a long time without realizing that these diseases are causing damage to their bodies. By being tested for these conditions, you will find out whether you may require further testing or treatment. In this way, you can seek appropriate advice and care to improve your health. This is a personal benefit to you.

The overall study results will be returned to and discussed by leaders and members of our community. These results may help our understanding of health and disease in our community and in South Africa. This could help us find new treatments or medical tests for disease in the future. This is unlikely to have any implications for you personally and you will not benefit financially from any new developments but it may ultimately benefit your community.

1. **How will my information and samples be used in the future?**

**Storing data and samples for future studies**

Once we have done the research that we are planning for this project, we would like to store your blood, samples and health information because they may be able to help scientists learn more in the future as new scientific questions emerge and new scientific techniques are invented. Your information, samples and results will be stored separately from your name and any other traceable information to ensure your confidentiality. A number code will be assigned to you and the link between your name and the number will be kept in an encrypted very secured format.

If you allow your samples and information to be stored, we may be able to find out things that will help improve our understanding of health and disease in your community in the future. If we find something that is of interest to the researchers, then we may contact you and invite you to participate in further research. If this occurs the future research will be ethically approved and will be explained to you in detail so that you can consider whether or not you wish to participate.

1. **Who is organising and funding the research?**

The study is being carried out by the Africa Health Research Institute in South Africa. The study is being funded through the Wellcome Trust, together with funding from other research sources.

In recognition for the time spent and the inconvenience experienced by participants of this study, the Africa Health Research Institute will provide reimbursement in the form of a food voucher to people who participate.

1. **Who has reviewed the study?**

This study has been ethically reviewed and approved by the Biomedical Research Ethics Committee at the University of KwaZulu-Natal in Durban, the London School of Hygiene Tropical Medicine in the UK, and Partners (Massachusetts General Hospital) in the US.

1. **Do I have to take part?**

No. You do not have to take part in this study. You can change your mind about taking part in the study at any point and you can withdraw your consent if you wish.

Please be aware that we will be able to remove or destroy any data or samples already collected up to the time you ask to withdraw unless these data or samples have already been used for scientific reports or publications. Anonymous data that have been shared with other researchers will not be retrievable.

Non-participation or withdrawal from the study will not affect the medical care you receive in any way except for the medical services (as chest X-ray and blood pressure measurements) that we provide through the study.

1. **Whom can I contact if I have questions or complaints about the study?**

We would like to answer all your questions. If you have any questions now, please ask us. If you have any questions or concerns about this study in the future, you can contact:

**Coordinator Community Engagement Unit**

Miss Ncengani Mthethwa

Africa Health Research Institute

Tel: 035 550 7500

Email: [nmsane@ahri.org](mailto:nmsane@ahri.org)

If you have any concerns about your rights in this study, please contact:

**BIOMEDICAL RESEARCH ETHICS ADMINISTRATION**

Research Office, Westville Campus

Govan Mbeki Building

Private Bag X 54001
Durban
4000

KwaZulu-Natal, SOUTH AFRICA

Tel: 27 31 2604769 - Fax: 27 31 2604609

Email: [BREC@ukzan.ac.za](mailto:BREC@ukzan.ac.za)

1. **Will you contact me in the future?**

We would like to contact you in the future for possible new studies related to this study. If this happens, you will be provided with full information about these additional studies. You are under no obligation to take part in future studies.

1. **Your consent**

If you want to take part in the study, you must complete a consent form to confirm your agreement. You will need to write your full name at the bottom of each form, then sign (or give a thumb print) and date it. We will also sign and date the form. You will be given a copy to keep and we will securely store a second copy for the Biobank records.

**If the participant is under 18 years, indicate if they are emancipated or not**

**(NB: child is considered emancipated if they live in a child-headed household with no adults, is married or is a biological parent)**

**Not emancipated ------------> parental consent required**

**Emancipated ------------> parental consent waivered**

**If child emancipated, indicate how:**

**Child lives in a child-headed household with no adults**

**Child is married**

**Child is a biological parent**

**CONSENT TICK BOXES AND SIGNATURE SPOTS (Including on tablet) FOR:**

I am the parent/guardian of_____________________________________________________

and confirm that I have read and understood the consent information sheet and consent to my child’s participation in this study

**Parental/guardian consent to allow child**

**(15-17 year old) to participate in study and be Yes No**

**independently consented**

____________________ ______________________ _____________________

Parent/guardian’s name parent/guardian’s signature (*1) Date

(print)

**(*1) FOR PERSONS WHO CANNOT WRITE MARK WITH ‘X’**

**____________________ ______________________ _____________________**

**Witness’ name (print) (*2) Witness’ signature Date**

**(*2) WITNESS ONLY IN CASE IF THE PARENT/GUARDIAN CANNOT WRITE. WITNESS MUST BE NEITHER CHILD NOR STUDY STAFF**


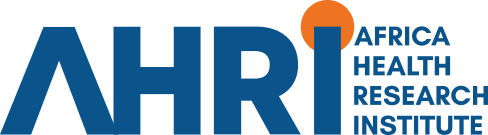


# *POP-C Vuk’uzazi*

# *Consent*

*Attach participant ID/barcode label*

### Declaration by participant:

By signing below, I …………………………………..…………………………………………… agree to take part in the above study entitled “POP-C/ Vukuzazi”. I understand the information sheet I have read, or have had read to me, and have been given a copy to keep. I have had the opportunity to discuss the study and ask questions, and I understand why the study is being done.

I declare that:

1. I am aged 18 years or older, and consent voluntarily to take part in this study and understand that my consent means full participation in the study to complete a survey and provide samples.
2. For the initial visit to the mobile clinic team I agree to:
   - participate in the questionnaire
   - have my height, weight, waist, hip and arm circumference, blood pressure and pulse measured for this study;
   - give a blood (for HIV and diabetes) and urine sample for research in the study and to be contacted for repeat sample collection if required for this study;
   - give a rectal swab

I understand that for significant health problems identified (such as diabetes and HIV) Vukuzazi will be able to give me a result. To ensure that I would like this information, a nurse will discuss each relevant test with me at the point that it is taken

1. For the initial visit to the mobile X-ray team I agree to:
   - participate in the questionnaire that looks for signs and symptoms of tuberculosis
   - have a chest X-ray taken (except for when I am pregnant)
   - submit a sputum sample, and if unable to produce a sputum undergo a procedure to make me cough
2. For my blood, sputum and urine samples, and rectal swabs I agree to:
   - to analyses that may provide information on diseases (such as possible anaemia and high cholesterol), information from which will not be relayed to me;
   - to genetic analyses, including DNA sequencing, to be undertaken in South Africa, or elsewhere, information from which will not be relayed to me. I understand that my genetic information will be made available to others, including via public databases of anonymised information, for future studies including for commercial purposes.
3. For the home follow-up visit by the nurse I agree to:
   - have a fingerprick for confirming diabetes (in case the initial measurement was elevated)
   - have a repeated blood pressure measurement (in case the initial measurement was elevated)
   - to be informed of my HIV status if I am newly diagnosed HIV positive, on the condition I gave consent for this
4. I agree to be contacted after referral to the DOH clinic to monitor linkage to care.
5. I agree for my name and contact details to be given to DOH if I am diagnosed with TB as part of this study.
6. I agree that other medical records as those from DOH and NHLS can be consulted and linked to this data.
7. I understand that only general study results will be returned to my community.
8. I understand that any research analysis or publication outcome will be based on anonymised data, that is, without my name, identifiable information or contact details attached.
9. I give permission for responsible individuals in the study teams in South Africa to look at the information gathered about me in previous surveys, where it is relevant to this study. I also understand that the study team will be able to access my samples, and my medical data through linked databases.
10. I understand that any information gathered about me and the samples I provide will be kept confidential, and stored anonymously and securely for future studies. I understand that future studies using my anonymised information or samples may be undertaken by other academic research institutions or commercial organisations in South Africa, the UK or other countries. I understand that the results of these future studies are unlikely to have any implications for me personally, and that I will not benefit financially if this research leads to the development of a new treatment or medical test.
11. I understand that I may be contacted in the future to discuss my experience in this study or for possible new studies. I also understand that I am under no obligation to take part in future studies.
12. I understand that my participation in this study is voluntary and that I am free to withdraw from the study, or any of its components, at any time without giving a reason and without this affecting my involvement in the rest of the study or my medical care or legal rights in any way.

Please tick box to indicate your consent

| Consent to take part in all study activities | Yes | No |
| --- | --- | --- |
| Consent to receive 50 Rand food voucher | Yes | No |
| Consent to have specimens stored | Yes | No |
| Consent to transfer my HIV, blood pressure, and diabetes results and my identifying information to the District Department of Health in the most secure manner possible | Yes | No |

---------------------------------------- ------------------- -----------------------------------------

Name of participant (Block letters) Date Signature or thumb print

**Declaration by person obtaining consent:**

I confirm that I have fully explained this research and provided complete and accurate information about it to ……………………………………………………….……………………………… and he/she has confirmed that he/she fully understands the information, as well as the risks and benefits associated with the study.

---------------------------------------- ------------------- -----------------------------------------

Name of person taking consent Date Signature

Name of witness (where participant could not read the consent form and it had to be read and interpreted)

---------------------------------------- ------------------- -----------------------------------------

Name of witness Date Signature

*One copy to be given to participant; one copy to be kept at local research site.*
